# A Novel Technique for Fetal ECG Extraction Using Single-Channel Abdominal Recording

**DOI:** 10.3390/s17030457

**Published:** 2017-02-24

**Authors:** Nannan Zhang, Jinyong Zhang, Hui Li, Omisore Olatunji Mumini, Oluwarotimi Williams Samuel, Kamen Ivanov, Lei Wang

**Affiliations:** 1 Shenzhen Institues of Adavanced Technology, Chinese Academy of Science, Shenzhen 518055, China; nn.zhang@siat.ac.cn (N.Z.); jyzhang@eee.hku.hk (J.Z.); hui.li1@siat.ac.cn (H.L.); omisore@siat.ac.cn (O.O.M.); samuel@siat.ac.cn (O.W.S.); kamen@siat.ac.cn (K.I.); 2 Shenzhen College of Advanced Technology, University of Chinese Academy of Sciences, Shenzhen 518055, China; 3 Department of Electrical and Electronic Engineering, The University of Hong Kong, Hong Kong 9990779, China

**Keywords:** adaptive noise cancellation (ANC), non-invasive FECG extraction, smooth window (SW), singular value decomposition (SVD), single abdominal channel

## Abstract

Non-invasive fetal electrocardiograms (FECGs) are an alternative method to standard means of fetal monitoring which permit long-term continual monitoring. However, in abdominal recording, the FECG amplitude is weak in the temporal domain and overlaps with the maternal electrocardiogram (MECG) in the spectral domain. Research in the area of non-invasive separations of FECG from abdominal electrocardiograms (AECGs) is in its infancy and several studies are currently focusing on this area. An adaptive noise canceller (ANC) is commonly used for cancelling interference in cases where the reference signal only correlates with an interference signal, and not with a signal of interest. However, results from some existing studies suggest that propagation of electrocardiogram (ECG) signals from the maternal heart to the abdomen is nonlinear, hence the adaptive filter approach may fail if the thoracic and abdominal MECG lack strict waveform similarity. In this study, singular value decomposition (SVD) and smooth window (SW) techniques are combined to build a reference signal in an ANC. This is to avoid the limitation that thoracic MECGs recorded separately must be similar to abdominal MECGs in waveform. Validation of the proposed method with r01 and r07 signals from a public dataset, and a self-recorded private dataset showed that the proposed method achieved *F1* scores of 99.61%, 99.28% and 98.58%, respectively for the detection of fetal QRS. Compared with four other single-channel methods, the proposed method also achieved higher accuracy values of 99.22%, 98.57% and 97.21%, respectively. The findings from this study suggest that the proposed method could potentially aid accurate extraction of FECG from MECG recordings in both clinical and commercial applications.

## 1. Introduction

Currently the most widespread techniques to monitor fetal health are Doppler ultrasound, invasive fetal electrocardiogram (FECG) monitoring, and non-invasive FECG monitoring. The monitoring method that adopts Doppler ultrasound is routinely applied during pregnancy and delivery [1]. However, the ultrasound technique has not been proven to be fully safe for the fetus [2]. In addition, the method does not allow for long-term monitoring since it is sensitive to maternal and fetal motion [3], and only provides one monitoring parameter that is the fetal heart rate (FHR), which is not sufficient for accurate fetal health monitoring. In the invasive FECG monitoring method, the FECG is recorded with the aid of electrodes attached to the fetal scalp while the cervix is dilated. With this method, monitoring can only be performed during delivery and the fetus would be prone to infection. In the non-invasive FECG monitoring method, FECG and MECG are collected simultaneously through electrodes attached to the mother’s abdomen, which could be performed at any point during the third trimester. Non-invasive FECG monitoring method could offer the FHR and FECG waveform morphology [3,4,5,6,7] that enables safe, accurate, and long-term overview of fetal well-being. This motivates researches in extracting FECG from AECG which contains the MECG, FECG, and noise. 

To date, a great deal of studies have proposed ways to extract the FECG for FHR monitoring. The first approach was the source separation method that attempted to separate MECG and FECG from AECG using spatial distribution [8,9], such as principal component analysis [10], independent component analysis [11,12], and periodic component analysis [13]. The above approaches aim to separate the underlying statistically independent sources into three components: MECG, FECG, and noise. The key assumption of the approaches is that of a linear stationary mixing matrix between these sources. The higher the number of available abdominal recordings, the better the FECG extraction. However, such large numbers of recordings would require the placement of several electrodes on the pregnant woman which could make them uncomfortable and as well make the procedure difficult to apply during activities of daily life. Consequently, the clinical use of the above approaches is quite limited due to the complex electrode configuration. The second set of approaches is the temporal methods that generate an estimation of MECG and the estimated MECG would be subtracted from AECG. Such methods are based on ANC, template subtraction (TS), and Kalman filter techniques among which, and the TS has been widely used in fetal heart rate (FHR) estimation [14,15]. However, the TS method is not good enough for removing abdominal MECG, which makes it difficult to locate the FECG R-peak accurately in the fetal heart rate (FHR) estimation. The methods [16,17] based on Kalman filter are also popular in FECG extraction using one abdominal recording. However, Kalman filters are limited by their computational complexity in long-term monitoring system. In addition, the Kalman filters sometimes fail when the MECG and FECG QRS waves entirely overlap [18]. Some recent studies also pay more attention on the preprocess procedure [19,20]. 

ANC is a classical method in FECG extraction that uses single channel thoracic MECG as reference signal, and one AECG as processed signal. ANC is based on training an adaptive filter to remove the projection of thoracic MECG on AECG recordings [11,12,21]. Therefore, the adaptive filter for abdominal MECG removal and FECG extraction require a reference signal that is morphologically similar to the abdominal MECG waveform [22,23]. The literatures [18,24] show that signal propagation from maternal heart to the abdomen is nonlinear and the morphology of the ECG waveforms (abdominal MECG and thoracic MECG) highly depends on the electrode locations. It is not always feasible to completely remove the abdominal MECG using the thoracic MECG as reference signal or even reconstructing the reference signal based on a linear combination of thoracic MECGs. Therefore, ANC is limited for fetal ECG extraction since a strict similarity between abdominal MECG and thoracic MECG is not always the case. To resolve this issue, [25] proposed the event synchronous adaptive interference canceller (ESAIC) concept as a specific application of ANC in MECG interference cancellation. The method attempted to use thoracic recording and abdominal recording to generate artificial signal which were used as reference. Deng et al. and Shao et al. have further studied the ESAIC concept. The method attempted to reduce the impact of nonlinear propagation from maternal heart to maternal abdomen on fetal ECG extraction to a certain extent [26,27]. Behar et al. also proposed a single-channel method that utilizes an echo state neural network based on ANC [28]. However, their approach should not be termed as single-channel algorithm, since it still requires a chest signal besides the abdominal channel.

In this paper, we propose an extensive ANC structure to remove the abdominal MECG from abdominal recording. Concretely, the smooth window (SW) technique and SVD method were combined (SWSVD) to estimate the abdominal MECG in the AECG signal. The estimated signal replaced the thoracic MECG recording and it was used as the reference signal in ANC. The limitation associated with the lack of strict waveform similarity between the interference (MECG in AECG) and the reference signal (MECG recorded in maternal thorax) could be avoided completely in the proposed method. The method only require one abdominal recording. Therefore, the electrode configuration allows for building light and comfortable recording systems for continuous long-term fetus monitoring. 

This paper is organized as follows: in Section 2, the theory and implemented detail of our proposed method are described. In Section 3, the two databases used in the current study and statistical assessment are presented. In Section 4, the theory and implementation detail of the four typical single-channel methods are presented in detail. Section 5 presents the Results while Section 6 discusses the results. Moreover, the paper is concluded in Section 7.

## 2. Materials and Methods

### 2.1. Basic Structure

**Figure 1:**
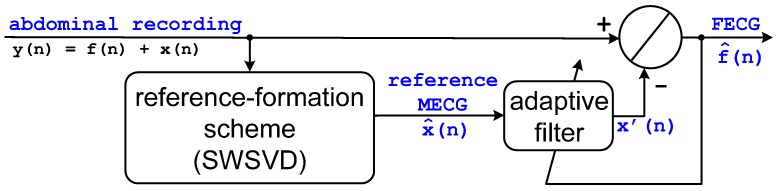
A block diagram of the proposed structure y(n): abdominal recording (AECG), *f*(*n*): FECG in AECG, *x*(*n*): MECG in AECG, $\hat{x}\left(n\right)$: reference MECG estimated by reference formation scheme (SWSVD), $\hat{f}(n$: extracted FECG).

The block diagram of the proposed method is depicted in Figure 1. The abdominal recording is considered to be AECG in this paper (the noise in the abdominal recording is ignored). The SWSVD scheme estimates the MECG of the abdominal signal that serves as input in form of the reference signal to the adaptive filter. The adaptive filter removes the MECG in the abdominal recording according to the estimated reference. For this purpose, an adaptive algorithm is built to model learning to minimize the interference signal (MECG) in abdominal recording with input $\hat{x}\left(n\right)$ and output $x^{\prime }\left(n\right)$, where $x^{\prime }\left(n\right)$ matches the target signal y(n) as closely as possible, and $\hat{f}\left(n\right)$ is the target signal and the extracted FECG represents the output of the adaptive filter.

### 2.2. Reference Formation Scheme SWSVD

**Figure 2:**
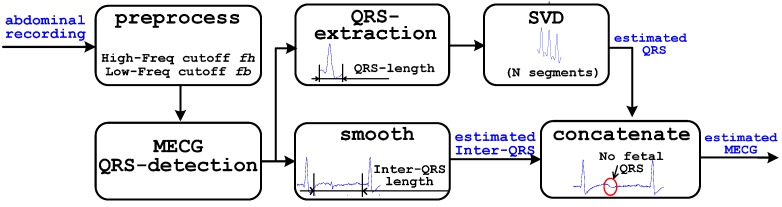
Block diagram of the SWSVD scheme.

A detailed description of the SWSVD scheme is presented in Figure 2. The aim of SWSVD is to create an artificial reference signal (estimated abdominal MECG) using a single channel abdominal recording. An ECG cycle basically consists of a P-wave, T-wave, and QRS complex (which can be subdivided into a separate Q-wave, R-wave, and S-wave) [29,30,31]. The T-wave, P-wave, and the segment between them are referred to as inter-QRS waveform in the current study. Basically, the estimation of the MECG from abdominal recording involves two steps which are dynamic estimation of MECG QRS segments using SVD method, and smoothening of FECG QRS in inter-QRS segments by average filter. The estimated and smoothed segments were concatenated as MECG wave which was used to build an artificial reference signal for the adaptive filter.

**Figure 3:**
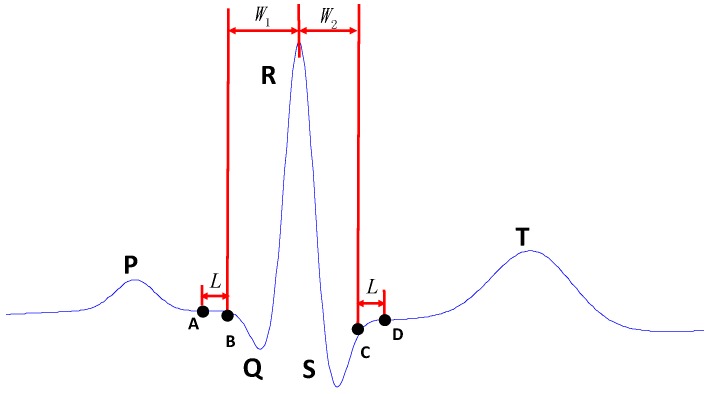
A representation of One AECG cycle.

The first step in estimation of MECG signals is to extract the AECG QRS complex from abdominal recordings. For this purpose, SVD method used in [32] was adopted. *N* preceding extracted MECG QRS complexes waves are arranged in the form of a designed matrix *Y*. The consecutive MECG QRS complex waveforms occupy consecutive rows of the later matrix, while the R peak lies in the same column. Hence, matrix *Y* is constructed as in Equation (1). Maternal R-peak was detected according the method proposed in the works of Pan and Tompkins [33]. The MECG QRS complex was extracted with fixed length before the maternal R-peak, *W*_1_, and after the maternal R-peak, *W*_2_, as shown in Figure 3:

(1) $$Y=\left[\begin{array}{ccc} y_{11} & \cdots & y_{1W}\\ \vdots & \ddots & \vdots \\ y_{N1} & \ldots & y_{NW} \end{array}\right]$$

where, *W* is the duration of AECG QRS cycle and *N* is the number of AECG QRS complex cycles:

(2) $$W=W_{1}+W_{2}+1$$

The MECG QRS complex component (principle components) of design matrix *Y* can be obtained using SVD. Parameter *N* was set to 20 in this study. The MECG QRS complex in the abdominal recoding can be estimated as follows:

(3) $$Y^{\prime }=\sum_{j=1}^{q}w_{j}w_{j}^{T}Y$$

where *q* is the principle component number of the abdominal recording and *W_j_* is the direction vector of the *j*-th principle component.

An appropriate reverse transformation is performed on the data reconstruction of the estimated MECG matrix *Y*’. Each data segment {*y*(·)} is transformed to {*x’*(·)}. As used in some earlier studies [30,31], the duration of the QRS complex segment is set and fixed as 0.1 s. In this current study, QRS wave segment is set to be the samples between 0.04 s and 0.06 s after the R peak was detected. In the later test section, *W*_1_ was set to be 40 sample points while *W*_2_ was set to be 60 sample points using a test data recorded at sample frequency of 1000 Hz. 

In the second step, a smoothening window method was applied to smooth the fetal component associated with the inter-QRS wave. In order to approximate the maternal inter-QRS waveform, an average filter was used to average a number of points from the inter QRS signal to produce each point in the output signal. In equation form, this can be expressed as:

(4) $$\hat{x}(i)=\frac{1}{M(i)}\sum_{j=-(M(i)-1)/2}^{j=(M(i)-1)/2}x(i+j)$$

where *x*(*i*) is AECG, $\hat{x}\left(i\right)$ is the estimated MECG, and *M*(*i*) is the number of points used in the moving average filter.

The group of points from the input signal is chosen symmetrically around the output point. Symmetrical averaging requires that *M*(*i*) be an odd number. If the sampling sequence *x*(*i*) is the sequence before point A and after point D of a previous cycle shown in Figure 3, then *M*(*i*) is set to be a constant *L* which is approximately equal to the length of FECG QRS complex, that is the number of sample points. The length of FECG QRS complex is about 26–61 ms when the pregnancy is between 17 to 41 weeks [34]. In this study, L is set to 30 samples in a test data with frequency of 1000 Hz. 

Jitter in the reference signal introduced by mismatch in the estimated QRS and Inter-QRS will cause a significant output error. In order to avoid such error in the estimated reference signal, the length of the average filter was reduced linearly when the input signal *x*(*i*) was spanning point A to B or point D to C.

That is to say, if AECG of *x*(*i*) is in the segment between points A and B or the segment between points C and D, the *M*(*i*) is determined by the following rules:

(5) $$M(i)=\left\{\begin{array}{c} \begin{array}{cc} i+L-k-W_{2} & k+W_{2}-L+1\leq i\leq k+W_{2} \end{array}\\ \begin{array}{cc} -i+L-W_{1}+k+1 & k+1-W_{1}\leq i\leq k+1-W_{1}+L-1 \end{array} \end{array}\right\}$$

where *i* is the location of AECG *x*(*i*); *k* and *k* + 1 represent the two adjacent R peak locations.

### 2.3. Adaptive Filter

For the adaptive filter, we adopted the recursive least square (RLS) method. In contrast to the least mean squares filter that adapts its coefficients by considering only the current error value, the RLS filter considers the total error from the initially supplied signal values to the current data point. In the context of FECG component extraction, the RLS filter tends to converge faster and is usually more accurate. The operational procedures of the RLS method are described as follows:

Let $\hat{x}\left(n\right)=\left[u\left(n-1\right),u\left(n-2\right),\ldots u\left(n-H\right)\right],$ be the input signal vector and $\omega \left(n\right)=\left[\omega_{1}\left(n\right),\omega_{2}\left(n\right),\ldots ,\omega_{H}\left(n\right)\right]$, be the coefficient vector of the adaptive filter. *H* is the number of filter coefficients and the output of the filter is given by:

(6) $$x^{\prime }(n)=\omega^{T}(n)\hat{x}(n)$$

The observable error is determined by using Equation (7):

(7) $$e(n)=y(n)-\omega^{T}\hat{x}{\left(n\right)}^{\prime }$$

In addition, the cost function was minimized by using the formulae presented in Equation (8):

(8) $$\varepsilon (n,\omega )=\sum_{i=1}^{n}\lambda^{n-i}{\left|e(n)\right|}^{2}$$

where λ∈[0,1] and it denotes the forgetting factor which determines the weight of previous data when updating filter coefficients.

To minimize the cost function *ε*(*n*), partial derivatives for all the entries of the coefficient vector were taken and results were initialized to zero as shown in Equation (9):

(9) $$\frac{\partial \varepsilon (n,\omega )}{\partial \omega }=0$$

At each iteration, the filter coefficients of the RLS algorithm were updated based on the following equations:

(10) $$\omega (n)=\omega (n-1)-R^{-1}(n)\overset{⌢}{x}(n)e(n)$$

(11) $$R(n)=\sum_{i=1}^{n}\lambda^{n-i}\overset{⌢}{x}(n){\overset{⌢}{x}}^{T}(n)$$

(12) $$R(n)=R(n-1)+\overset{⌢}{x}(n){\overset{⌢}{x}}^{T}(n)$$

The formula in Equation (12) was derived based on Equations (10) and (11):

(13) $$R^{-1}(n)=R^{-1}(n-1)-\frac{R^{-1}(n-1)\hat{x}(n){\hat{x}}^{T}(n)R^{-1}(n-1)}{1+{\hat{x}}^{T}(n)R^{-1}(n-1)\hat{x}(n)}$$

The parameters *λ* and *H* were set to 0.99 and 20, respectively, in the study.

## 3. Database and Statistical Assessment

### 3.1. Database

Two different datasets were used in the current study to assess the performance of the proposed method. The first dataset was collected from an online clinical database (Abdominal and Direct Fetal ECG Database [35]) provided by the PhysioNet. The Abdominal and Direct Fetal ECG Database consist of five multichannel datasets, that is: r01, r04, r07, r08 and r10, recorded during delivery processes. Each multichannel dataset consists of four AECG recordings acquired from the maternal abdomen and one direct FECG recording acquired from the fetal head simultaneously. The configuration of the abdominal electrodes comprised of four electrodes placed around the navel, a reference electrode placed above the pubic symphysis, and a common mode reference electrode (with active-ground signal) placed on the left leg. In order to reduce the skin impedance, the areas under the Ag-AgCl electrodes (Red Dot 2271, 3M, St. Paul, MN, USA) were abraded. The first AECG recording of r01 and the fourth AECG recording of r07 are termed as public dataset and denoted as DB1 in this study. 

**Figure 4:**
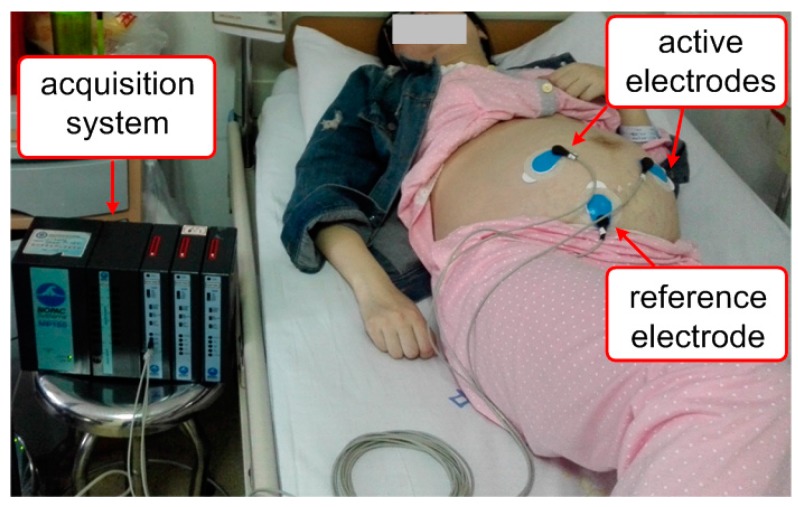
Experimental settings for capturing DB2.

Meanwhile, the second set of data was obtained with BIOPAC Acquisition Systems, Inc. (Goleta, CA, USA), provided with a ECG100C module at the PLA Navy Anqing Hospital, Anhui, China. The acquisition procedures includes placing the two active electrodes below the mother’s navel, thus to be as closer as possible to the heart of the fetus as in Figure 4. The electrodes were of type “offset-electrode” and this was chosen to ensure high-quality recording [36]. This dataset contains a single abdominal recording that was captured from the abdomen of a pregnant woman at 40 weeks of gestation. The signal were sampled at 1000 Hz and the private database is denoted DB2.

Both the public and private datasets were used to evaluate the performance of the proposed method and the four typical single channel FECG extraction methods considered in this study as shown in Section 5.

### 3.2. Statistical Assessment

Statistical analysis was conducted to compare the performance of the proposed method with four existing typical single channel methods in terms of FECG extraction accuracy. The adapted version [37] of the Pan and Tompkins algorithm [33] was used to detect the fetal QRS in this study. The performance of the FECG QRS complex detection was assessed by beat-to-beat comparisons between the detected FECG QRS complex and the annotated. In accordance with the ANSI/AAMI guideline [38], sensitivity (*Se*), positive predictive value (*PPV*), and accuracy (*ACC*) metrics [39] were used for the assessment and there formulae are presented below:

(14) $$Se=\frac{TP}{TP+FN}$$

(15) $$PPV=\frac{TP}{TP+FP}$$

(16) $$ACC=\frac{TP}{TP+FP+FN}$$

where *TP*, *FP* and *FN* are the number of true positives (correctly detected FECG QRS complex), false positives (falsely detected non-existent R peaks), and false negative (missed FECG QRS complex detections) respectively. *T* = *TP* + *FN* is the number of annotated FECG QRS complex. The *F1* is the overall probability that the FECG QRS complex are detected correctly and could alternatively be used as a measure of the method’s accuracy:

(17) $$F_{1}=2\frac{PPV•Se}{PPV+Se}=\frac{2•TP}{2•TP+FN+FP}$$

The adult ECG acceptance interval between the detected and annotated locations of QRS complex was defined as 150 ms [40], and a matching interval of 50 ms was used in the FECG QRS complex detection due to higher FHR [41]. PhysioNet provides an implementation of FECG QRS detection which is available online [42]. In the implementation, fetal heartbeats were detected with an energy threshold of 0.42 and the refractory period between two R-peaks is 0.15 s.

## 4. Compared Methods

Four typical single-channel methods were implemented in order to evaluate performance of the proposed method. Procedures of the four methods are discussed briefly in this section.

### 4.1. Cerutti Method

The Cerutti method proposed in [43] builds a maternal template to each cycle by averaging and scaling procedures. Afterwards, the maternal templates are subtracted from each abdominal cycle, leaving residual FECG and noise. As presented in other studies [30,31], the P wave, QRS complex and T wave durations are normally about 0.2 s, 0.1 s and 0.4 s, respectively. The maternal templates are defined as signals from 0.25 s before the detected maternal R-peak and 0.45 s after the R peak. An averaging template $\vec{x}$ is then made as mean of N preceding maternal templates synchronized on mother R peak and this is updated on every maternal cycle. The parameter N is set to 20 in our implementation of this method. Subsequently, the averaging template is scaled with a constant factor *γ*. Hence, the estimated maternal template is expressed as $\vec{\hat{x}}=\gamma \vec{x}$. Then, the estimated maternal template $\vec{\hat{x}}$ is subtracted from the abdominal cycle $\vec{y}$. Scaling factor γ is based on the search mechanism of the least-mean square (LMS) error *e*^2^ between $\vec{\hat{x}}$ and $\vec{y}$.

(18) $$e^{2}=\min\left\|\gamma \vec{x}-\vec{y}\right\|$$

Since maternal and fetal QRS are not synchronized, and fetal QRS is larger than maternal QRS in one order amplitude, no FECG is present in $\vec{\hat{x}}$. Therefore, the FECG is left when estimated template $\vec{\hat{x}}$ is subtracted from abdominal cycle $\vec{y}$.

### 4.2. Kanjial Method

The Kanjial method, as described in [32], use SVD to estimate principle components related to MECG in an abdominal recording. The estimated MECG is cancelled from the abdominal recording to produce FECG. The abdominal sample data is arranged as the in a design matrix *X* such that consecutive MECG cycles occupy consecutive rows of the matrix, and peaks of the MECG cycles lie in the same column of the matrix:

(19) $$X=\left[\begin{array}{ccc} x_{11} & \cdots & x_{1t}\\ \vdots & \ddots & \vdots \\ x_{p1} & \ldots & x_{pt} \end{array}\right]$$

where *m* and *n* are the number of MECG cycles and samples sizes in MECG cycle, respectively.

SVD is performed on design matrix *X* and the most dominant component $X_{mother}=U\sum V^{T}$ is the estimated maternal component, forming the fetal component $X_{fetus}=X-X_{mother}$, where $U\in {ℜ}^{pxp},\;V\in {ℜ}^{txt},\;U^{T}U=I,\;V^{T}V=I,\;\sum =\left\{diag\left(\sigma_{1},\ldots ,\sigma_{g}\right)\ldots 0\right\}$, *g* = min(p,t).

The design matrix is updated every 20 MECG cycles and the duration of MECG cycles is 0.7 s (0.25 s before and 0.45 s after the detected maternal R peaks).

### 4.3. Suzanna Method

The Suzanna method [30] is an improvement of Cerutti method. In the estimating maternal ECG procedure, Cerutti method scale the average maternal ECG template as a whole while Suzanna method performs a separate scaling of the P wave, the QRS complex and the T wave. Refer to the paper [30], the maternal template $\vec{x}$ is windowed with a total length of 0.7 s. In this method, QRS wave segment is the samples within 0.05 s before and after the detected R peak. P wave segment and the T wave segment is the samples within 0.2 s before and 0.4 s after the QRS wave segment. The segments vectors are assembled in a matrix *M* as follows:

(20) $$S=\left(\begin{array}{ccc} \vec{x_{P}} & 0 & 0\\ 0 & \vec{x_{QRS}} & 0\\ 0 & 0 & \vec{x_{T}} \end{array}\right)$$

The scaling parameters $\gamma_{P},\;\gamma_{QRS},\;\gamma_{T}$ correspond to the P wave segment, T wave segment and QRS wave segment. The scaling vector:

(21) $$\vec{\gamma }=\left(\begin{array}{ccc} r_{P} & r_{QRS} & r_{T} \end{array}\right).$$

The estimated maternal template is given by $\vec{x^{\prime }}=S\vec{r}$. The scaling vector is solved based on the search of min least squares error $minS\vec{r}-\vec{y}$:

(22) $$\vec{r}={\left(S^{T}S\right)}^{-1}S^{T}\vec{y}$$

In the averaging procedure, the averaging maternal template is built using 20 maternal cycles and updated on every maternal cycle.

### 4.4. Vullings Method

The Vullings method, proposed in [3], only estimate the MECG by linear combination of preceding MECG cycles with different weight coefficients. *X* is the prediction matrix and synchronized on maternal R peaks.

(23) $$X=\left[\begin{array}{ccc} x_{11} & \cdots & x_{1n}\\ \vdots & \ddots & \vdots \\ x_{m1} & \ldots & x_{mn} \end{array}\right]$$

where *m* and *n* are the number of MECG cycles and sample sizes in cycle. The MECG cycle vector $\vec{x}$ could be estimated by the prediction matrix *X* and weighted coefficient vector $\vec{\lambda }=\left[\lambda_{1}\lambda_{2}\ldots \lambda_{m}\right]$ as in Equation (24):

(24) $$\vec{\hat{x}}=\vec{\lambda }X^{T}$$

The weight coefficient vector is calculated by minimizing the mean squared error (MSE) between the estimated MECG cycle vector $\vec{\hat{x}}$ and actual MECG cycle vector $\vec{x}$ as in Equation (25):

(25) $$\vec{\lambda }=(X^{T}X{)}^{-1}X^{T}\vec{x}$$

The parameter *m* is set to seven maternal cycles and the duration of MECG cycles is 0.7 s, that is, 0.25 s before and 0.45 s after the detected maternal R peaks.

## 5. Results

The proposed method was implemented in Matlab 8.6.1 (R2015b) on a computer equipped with a 2 GHz Pentium^®^ processor. This system configuration is capable of removing MECG from the abdominal signals in real time.

### 5.1. Preprocess

**Figure 5:**
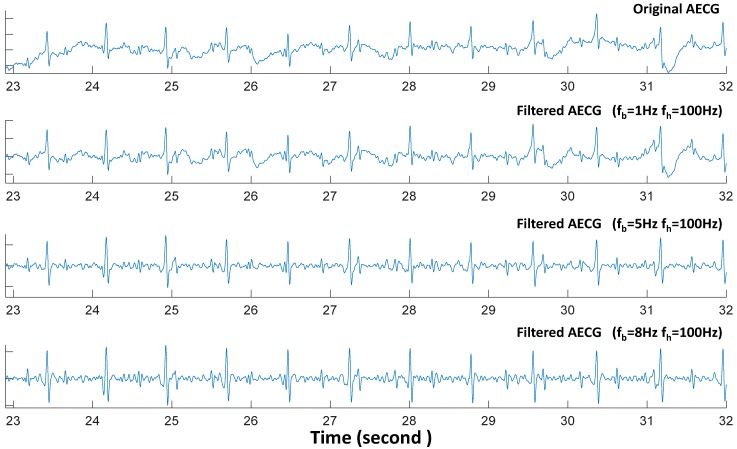
The different preprocessed AECG wave with different baseline cut-off frequency *f_b_* and the same high frequency *f_h_* = 100 Hz.

In this study, five-order low-pass and five-order high-pass Butterworth bidirectional filters were used to remove the high frequency and low frequency signals, respectively. Figure 5 shows the different filtered results for one segment of r01 recording in DB1 using three choices of the low cut-off frequency, namely 1, 5 and 8 Hz. In all cases, the same high cut-off frequency of 100 Hz was used.

### 5.2. Performance Based on DB1

**Figure 6:**
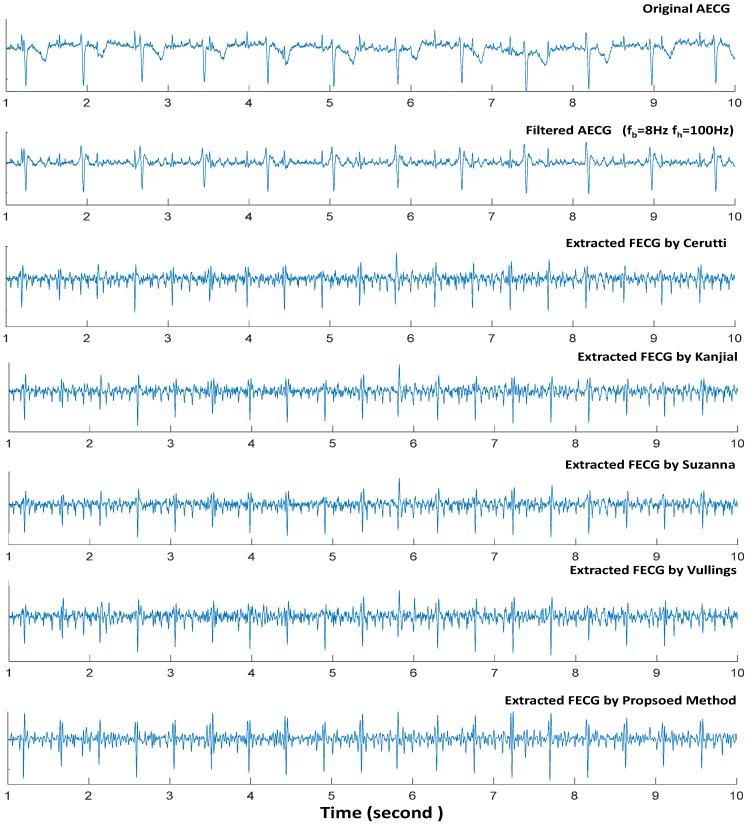
A segment performance using signal r01 (DB1) of our proposed method and other four typical single-channel methods with same preprocessed (low cutoff frequency *f_b_* = 8 Hz and high cutoff frequency *f_h_* = 100 Hz).

DB1 contains two signals: signal r01 and signal r07. The r01 signal has a duration of 5 min with a total of 642 fetal heartbeats while signal r07 also has a duration of 5 min but a total of 625 fetal heartbeats. Both signals were preprocessed with the same condition, that is, low cutoff frequency (*f_b_*) of 8 Hz and high cutoff frequency (*f_h_*) of 100 Hz. This was done to evaluate the performance of the proposed method and the four typical single-channel FECG extraction methods. Table 1 and Table 2 show the FECG QRS detector statistical assessment for signals r01 and r07, respectively. Direct FECG channels in DB1 provide the fetal heart location accurately. Figure 6 shows a single segment performance using signal r01 of our proposed method and four typical single-channel methods with same preprocessing procedure, that is, low cutoff frequency (*f_b_*) of 8 Hz and high cutoff frequency (*f_h_*) of 100 Hz.

### 5.3. Performance Based on DB2

**Figure 7:**
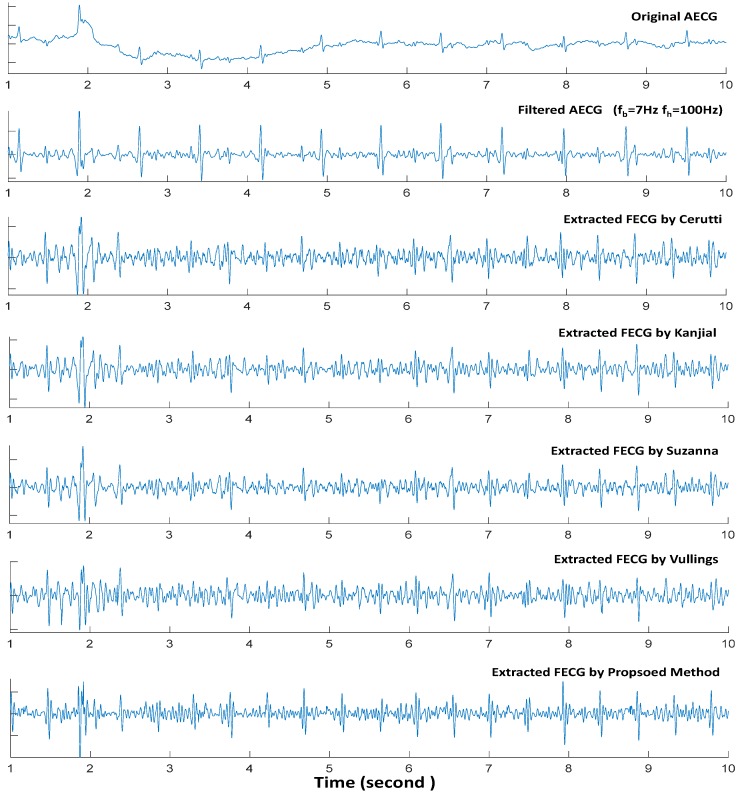
A segment performance using DB2 of our proposed method and other four typical single-channel methods with same preprocessed (low cutoff frequency *f_b_* = 7 Hz and high cutoff frequency *f_h_* = 100 Hz).

Figure 7 presents one segment performance using the private database (DB2). The duration of AECG recordings in the database is 1 min 20 s, and it contains a total of 177 fetal heartbeats. Locations of the fetal heartbeats in the dataset from DB2 were labeled by two doctors in the PLA Navy Anqing Hospital, Anhui, China. The database was used to test the performances of the proposed method and other four typical single-channel methods explained in previous section. Table 3 is the FECG QRS detected from statistical assessment based on DB2. The results presented in Table 3 and Figure 7 are based on the same preprocessing condition (low cutoff frequency *f_b_* = 7 Hz and high cutoff frequency *f_h_* = 100 Hz).

## 6. Discussion

The pre-processing is a crucial step in FECG extraction from abdominal recordings. The low frequency cut-off recommended is 0.05 Hz for the diagnostic electrocardiographs [44]. In the context of non-invasive FECG extraction, it is common to use a low cut-off frequency value of more than 0.05 Hz. Figure 5 shows the different abdominal ECG waves with different baseline cut-off frequency *f_b_*. It is obvious that some FECG QRS is hardly visible at very low amplitude or overlaid totally by noise in the original AECG. The FECG QRS have been enhanced by a low frequency cut-off which is greater than 5 Hz. It is reasonable that such low cut-off frequency affects the morphology of the ECG signals but this is acceptable since the objective is to analyze FHR [45]. For all approaches, higher *f_b_* (*f_b_* = 10 Hz) have been shown to perform better than a lower *f_b_* (*f_b_* = 2 Hz) with up to 3% increase with respect to *F1* measure [3].

In order to maximally suppress the artifacts and minimize distorted FECG signal, preprocessing conditions with low (*f_b_* = 8 Hz) and high (*f_h_* = 100 Hz) cut-off frequencies were used to the dataset obtained from DB1. Meanwhile the dataset from DB2 was preprocessed with low (*f_b_* = 7 Hz) and high (*f_h_* = 100 Hz) cut-off frequencies. 

Figure 6 shows an example of a single segment with the proposed method and other typical single-channel methods by using signal r01 obtained from DB1. The performance of our FECG extraction method and the existing methods was assessed from two aspects of the segment. The first aspect shows the ability of the methods to extract the fetal QRS component. It was observed that the fetal heartbeat was successfully extracted even though the fetal QRS overlapped with the maternal QRS. In the second aspect, the ability of the methods with respect to removing the MECG QRS complex was shown. From both aspects, the proposed method was observed to have better performance than the existing methods by removing the maternal QRS from the AECG recordings. In addition, the maternal QRS component could not be cancelled completely, and this would make it difficult to accurately discriminate the fetal QRS. As shown in Figure 6, all the five single-channel FECG extraction methods performed well. However, our proposed method have the best performance in removing MECG component. From Table 1, it could be observed that our proposed method only recorded two false positive fetal heartbeats detection and three false negative fetal heartbeats detection based on signal r01 which contains a total of 642 fetal heartbeats. Suzanna method achieved an *F1* score of 99.53% at low and high cut-off frequencies of *f_b_* = 8 Hz and *f_h_* = 100 Hz respectively, while our proposed method recorded a slightly higher F1 score of 99.61%. Also from Table 2, the proposed method only recorded four false positive fetal heartbeats detection and five false negative fetal heartbeats detection based on signal r07 which contains a total of 625 fetal heartbeats. Hence, our proposed method has the best performance in fetal heartbeats detection using the signals in DB1. 

The DB2 just contain one channel AECG and the signal length is 1 min and 20 s. A total of 177 fetal heartbeats is contained in the DB2. Compared to DB1, the FECG QRS is interfered by a lot of noise which is very close with FECG QRS in frequency domain. In addition, the morphology of the MECG QRS complex displays time-variation and amplitude-variation largely. That is to say, signal quality of DB2 is much worse than the signals in DB1. From Figure 7, it is obvious that the MECG QRS component cannot be removed completely by the four existing single channel methods considered in this study. The residual MECG components have severely affected the identification of beat locations in the fetal ECG. The FECG QRS statistical assessment in Table 3 also indicates that the residual MECG QRS component resulted to more false positive and false negative fetal heartbeats detection in DB2. However, the F1 of our proposed method is 0.9858 while the F1 of Cerutti, Kanjial, Suzanna, and Vullings methods are 94.65%, 94.89%, 92.35% and 82.62%, respectively. It is obvious that our proposed method outperformed other single-channel FECG extraction methods with respect to DB2. One possible reason why the proposed method performed better than the existing single channel methods is that it integrates an extensive ANC structure that can effectively cancel the interferences in terms of MECG in the AECG. Therefore, when dealing with signals of poor quality (like DB2), our proposed extensive ANC structure could be effective during FECG extraction.

The four methods (Cerutti, Kanjial, Suzanna, and Vullings) that were considered for comparison in this study rely on the construction of an adaptive MECG template using the preceding MECG cycle. The estimated MECG template is subtracted coherently from AECG and FECG is left. These temporal methods have been widely used in literatures. Our work focuses on such methods because they only require a relatively low amount of dataset (a single abdominal channel) and are of low computational complexity that would allow long-term monitoring. However, the statistical analyses based on DB2 showed that the existing methods could not remove the MECG QRS component from AECG completely in situations where there are large variation in the morphology of the continuous MECG QRS complexes. The proposed extensive ANC structure performed better than the four methods in estimating MECG using continual preceding MECG QRS waveforms. Therefore, the proposed extensive ANC structure using average window technique and SVD method could effectively estimate the MECG in FECG extraction for FHR.

## 7. Conclusions

In this study, a novel framework based on ANC technique that uses a single channel abdominal recording for FECG extraction is proposed. The proposed method integrates averaging window and SVD techniques to build reference signal for the ANC based extraction framework. The proposed method could avoid the limitation of recording at least one thoracic MECG which should be linear with an abdominal MECG recording, and this is common with the classical ANC methods. Datasets consisting of abdominal and direct fetal ECG from two different sources were used to assess the performance of the proposed system and four typical single channel methods. Compared with the four single-channel methods, the proposed method performed better in terms of ACC and F1 metric. Also, the statistical results shows that the proposed method is able to produce reliable fetal heartbeat extraction using a single abdominal recording.

## Figures and Tables

**Table 1:** FECG QRS detected from statistical assessment of signal r01 (DB1).

Methods	*F1* (%)	*PPV* (%)	*SE* (%)	*ACC* (%)	*TP*	*FP*	*FN*
Cerutti	0.9945	0.9953	0.9938	0.9891	638	3	4
Kanjial	0.9922	0.9938	0.9907	0.9845	636	4	6
Suzanna	0.9953	0.9953	0.9953	0.9907	639	3	3
Vullings	0.9890	0.9937	0.9844	0.9783	632	4	10
**Proposed Method**	**0.9961**	**0.9969**	**0.9953**	**0.9922**	**639**	**2**	**3**

**Table 2:** FECG QRS detected from statistical assessment of signal r07 (DB1).

Methods	*F1* (%)	*PPV* (%)	*SE* (%)	*ACC* (%)	*TP*	*FP*	*FN*
Cerutti	0.9896	0.9873	0.9920	0.9795	620	8	5
Kanjial	0.9880	0.9872	0.9888	0.9763	618	8	7
Suzanna	0.9912	0.9889	0.9936	0.9826	621	7	4
Vullings	0.9689	0.9666	0.9712	0.9396	607	21	18
**Proposed Method**	**0.9928**	**0.9936**	**0.9920**	**0.9857**	**620**	**4**	**5**

**Table 3:** FECG QRS detected from statistical assessment of DB2.

Methods	*F1* (%)	*PPV* (%)	*SE* (%)	*ACC* (%)	*TP*	*FP*	*FN*
Cerutti	0.9465	0.9438	0.9492	0.8984	168	10	9
Kanjial	0.9489	0.9543	0.9435	0.9027	167	8	10
Suzanna	0.9235	0.9261	0.9209	0.8579	163	13	14
Vullings	0.8262	0.8333	0.8192	0.7039	145	29	32
**Proposed Method**	**0.9858**	**0.9886**	**0.9831**	**0.9721**	**174**	**2**	**3**
